# Aurora‐Associated Geomagnetic Activity and Health: A Review With Focus on Neurological Implications

**DOI:** 10.1029/2025GH001710

**Published:** 2026-06-22

**Authors:** Yashendra Sethi, Vidhi Vora, Amogh Verma, Rahul Bhatt, Manjeet Singh Chaudhary, Hitesh Chopra, Nigel H. Greig

**Affiliations:** ^1^ PearResearch & National Institute of Cardiometabolic Excellence (NICE) Vigyaved Healthcare Dehradun Uttarakhand India; ^2^ Subharti Medical College Swami Vivekanand Subharti University Meerut India; ^3^ Department of Internal Medicine Rama Medical College Hospital and Research Centre Hapur India; ^4^ SMS Medical College Jaipur India; ^5^ Department of Biosciences Saveetha School of Engineering Saveetha Institute of Medical and Technical Sciences Chennai India; ^6^ Translational Gerontology Branch Intramural Research Program National Institute on Aging National Institutes of Health Baltimore MD USA

**Keywords:** geomagnetic activity, auroras, neurological health, neurosurgery, seizure frequency, cognitive function, postoperative complications

## Abstract

Geomagnetic activity (GMA), particularly during auroras, has emerged as an intriguing area of research due to its potential health impact. Prior studies indicate that fluctuations in geomagnetic fields can affect diverse physiological and psychological outcomes. The aim of this article is to comprehensively review the impact of GMA on neurological health with an emphasis on implications for patients potentially undergoing a neurosurgical procedure or neurologic/psychiatric assessment. A comprehensive literature review was therefore performed, examining peer‐reviewed articles sourced from PubMed and Google Scholar. The focus was on evaluating potential correlations between geomagnetic disturbances (GMDs) and a range of health outcomes, particularly in relation to neurosurgical, neurological and neuropsychiatric contexts. Reported associations included: increased seizure frequency in patients with epilepsy, sleep disturbances affecting recovery, and cognitive impairments that may complicate patient consent processes. Additionally, heightened stress and anxiety levels during geomagnetic storms could pose challenges for patient management in surgical settings. This review underlines that such disturbances can potentially result in significant postoperative complications, particularly in the elderly, necessitating enhanced monitoring and tailored care strategies. Understanding the diverse effects of GMA on health is essential for optimizing patient outcomes, particularly in surgical procedures. This review highlights the need for further research to elucidate underlying GMA‐triggered molecular mechanisms and establish evidence‐based guidelines that consider geomagnetic conditions in neurological/neuropsychiatric evaluations, surgical planning and postoperative care, especially for vulnerable populations. An enhanced awareness among neurosurgeons, psychiatrist, neurologist and healthcare providers is essential to mitigate potential adverse effects of GMA on patient health and optimize recovery.

## Plain Language Summary

Geomagnetic storms derive from temporary disturbances of the earth's magnetosphere that are driven by solar interactions and often are associated with visually spectacular exhibitions of colorful light in the Earth's atmosphere–termed the “Northern and Southern Lights” (auroras). These storms and their associated auroras are principally observed in high‐latitude regions (around the Arctic and Antarctic circles). However, recently they are being routinely witnessed across northern Europe, North America, and parts of the Southern Hemisphere, including New Zealand, South Africa and South America. Our review article evaluates the available scientific literature–with a focus on neurological perspectives, the need for further research to elucidate underlying mechanisms and to establish evidence‐based guidelines that consider geomagnetic conditions in neurological/neuropsychiatric evaluations, surgical planning and postoperative care, particularly for vulnerable populations like the elderly. Previous studies have indicated a link between geomagnetic activity and various health outcomes, including an increase in seizure activity and mood disorders. However, comprehensive literature evaluations addressing the impacts on neurological and neurosurgical patients are lacking. This review provides a critical examination of the implications of geomagnetic activity on neurological health and surgical outcomes, emphasizing the need for awareness among healthcare providers to optimize patient management.

## Key Points

- Geomagnetic disturbances are associated with measurable changes in autonomic, neuroendocrine, and neurological parameters across observational studies
- Variations in geomagnetic activity have been linked to seizure frequency, cognitive fluctuations, and mood‐related outcomes in susceptible populations
- Current evidence remains largely associative, highlighting the need for mechanistic and longitudinal studies to clarify clinical relevance

## Graphical abstract

Health Impacts of Aurora‐Related Geomagnetic Activity. *Image sources*: segments of the figure were created using visuals from Wikipedia Commons, Pixabay, Flaticons, Pikbest, Pxhere, Publicdomainvectors, Openclipart, and Servier Medical Art, all licensed under Creative Commons.

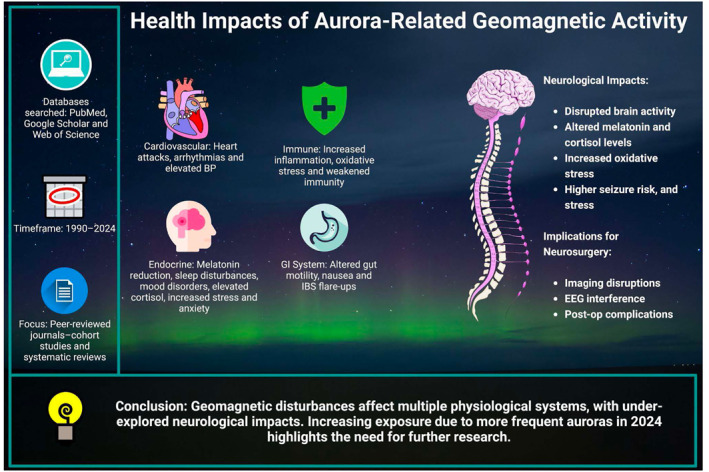

## 1 Introduction

Auroras are visually stunning natural light displays, typically seen near polar regions, that are caused by the interaction of solar winds with Earth's magnetosphere. Such interactions produce geomagnetic disturbances (GMDs), a phenomenon that has increasingly gained scientific attention due to their potential health effects on multiple systems, including the neurological, cardiovascular, endocrine, immune, and gastrointestinal systems. In 2024, auroras became more visible far from the poles in regions such as Europe, North America, and even parts of the Southern Hemisphere, including New Zealand and South Africa ‐ with the US National Aeronautics and Space Administration noting that the 2024 auroras were some of the strongest in the past 500 years (Roush, 2024; Will, 2024 Be the Year of the Aurora? ‐ British Geological Survey, n.d.; Zilli Vieira et al., 2019). In addition to the potential of such storms disrupting technology on earth–from satellites and GPS to radio communications and the power grid (Treisman & Khurana, n.d.)–they lead to an increased exposure to geomagnetic activity (GMA) in populations that are not accustomed to such phenomena (Zilli Vieira et al., 2019). This growing frequency of auroras worldwide signals a question as to whether GMDs may have a broader and more profound effect on public health than previously considered? And makes this a timely topic for review. For clarity, “auroral geomagnetic activity” denotes disturbances associated with charged particle precipitation in the upper atmosphere that give rise to visible auroral emissions, whereas “geomagnetic activity” more broadly encompasses fluctuations in the Earth's magnetic field irrespective of optical manifestation. Auroras therefore represent epiphenomena—visual correlates of underlying GMDs—rather than independent biological exposures. Accordingly, the physiological effects discussed herein are attributed to GMDs and their associated electromagnetic field variations, with auroral displays serving solely as observable indicators of heightened GMA.

The health impacts of GMDs can potentially influence multiples systems. For instance, GMA has been linked to cardiovascular issues such as arrhythmias, ischemic heart disease, and stroke, particularly during periods of heightened solar storms (Feigin et al., 2014; Rezende et al., 2025; Shamyar & Yeghikyan, 2025; Vencloviene, 2013; Vencloviene et al., 2013). Multiple studies have documented a reduction in heart rate variability (HRV) alongside elevations in heart rate and blood pressure (BP), with lower HRV independently predicting increased mortality and sudden cardiac death risk (Sessa et al., 2018), and demonstrating graded reductions across low‐, intermediate‐, and high‐risk groups with discriminatory performance for SCD stratification (AUC ∼0.72) (Yan et al., 2023), thereby reflecting impaired autonomic dysregulation in susceptible individuals. Furthermore, GMDs have been reported to influence hormone regulation, particularly the production of melatonin and cortisol, which are crucial for sleep regulation, circadian rhythm maintenance, and the body's stress response (Weydahl et al., 2000). Resulting potential hormonal imbalances can affect the immune system, exacerbating inflammatory and autoimmune conditions (Close, 2012; Kiznys et al., 2020; Martel et al., 2023; Pierre & Persinger, 1998). GMDs can, additionally, potentially disrupt gastrointestinal functioning, leading to symptoms like nausea, altered digestion, and gut permeability, likely due to changes in the autonomic nervous system (ANS) (Sandyk, 1990).

Despite these widespread potential effects, a significant gap remains in understanding the neuropsychiatric and neurological consequences of GMDs. Whereas studies have explored cardiovascular and endocrine effects, the brain's susceptibility to GMDs and the associated risks for mood disorders, cognitive dysfunction, and neurodegenerative diseases have been under‐researched. This article aims to highlight these neuropsychiatric and neurological impacts, which are particularly concerning for vulnerable populations such as the elderly, individuals with psychiatric conditions, and those with neurodegenerative diseases. Moreover, as auroras become more common beyond polar regions (Roush, 2024; Will, 2024 Be the Year of the Aurora? ‐ British Geological Survey, n.d.; Zilli Vieira et al., 2019), the general population's exposure to GMDs is increasing, necessitating a broader examination of their impact.

## 2 Materials and Methods

To compile this review, a structured search of the scientific literature was conducted across PubMed, Google Scholar, and the Web of Science databases using specific keywords, such as “geomagnetic disturbances,” “auroras,” “health impacts,” and “solar storms.” Given the limited work published on the topic and the different study designs and populations (human and animals), the scientific literature available was insufficient to support a classical systematic review/meta‐analysis (with accompanying PRISMA flowchart), hence a narrative review of articles published between 1990 and 2025 was prioritized, with a focus on peer‐reviewed journal articles, cohort studies, and systematic reviews. Studies were included based on their relevance to health impacts across physiological systems, particularly in relation to findings on neurological and neuropsychiatric health. Data were synthesized qualitatively and discussed between the authors to provide a comprehensive overview of the impacts of GMA. For studies synthesized into Tables 1 and 2, the GRADE approach was used to evaluate supporting scientific evidence: Strong (i.e., high certainty): Evidence comes from multiple well‐conducted randomized clinical trials, meta‐analyses, or large cohorts with consistent results, minimal bias, and direct applicability. Moderate: Evidence downgraded due to limitations (e.g., risk of bias, inconsistency, indirectness, imprecision, or publication bias). The true effect is likely close to the estimate, but future studies could change the conclusion.

**Table 1:** Effects of Aurora‐Related Geomagnetic Disturbances (GMDs) on Various Bodily Systems

Body system	Effect of aurora‐related geomagnetic disturbances	Implications	Influencing risk factors/Effect modifiers and confounders	Level of evidence with current research	References
Cardiovascular System	Increased admissions for acute myocardial infarction and higher diastolic blood pressure during geomagnetic storms.	Higher morbidity and mortality rates; potential for increased cardiovascular events.	Pre‐existing cardiovascular conditions, age, gender.	Strong evidence from large epidemiological studies.	Stoupel (2002)
Nervous System	Increased incidence of psychiatric admissions and exacerbation of neuropsychiatric disorders during geomagnetic disturbances.	Heightened risk of mental health crises; potential impact on daily functioning.	Prior mental health conditions, occupational stressors, geographic location.	Moderate evidence; significant correlations noted but require causal analysis.	(Altpeter et al., 2006; Bhattacharyya et al., 2008; Bouché & McConway, 2019; Iashmanov & Koshelevskiǐ, 2008; Krylov, 2017; Martel et al., 2023; Milojević, 2016; Mohammed Ali, 2016; Park et al., 2007; Sumsuzzman et al., 2021; Tian et al., 2024; Zhang et al., 2017; Zilli Vieira et al., 2022)
Endocrine System	Altered levels of hormones (e.g., increased prolactin and corticosteroids) during periods of high geomagnetic activity.	Potential for dysregulation of metabolic processes and stress responses.	Individual hormonal baseline levels, stress exposure, environmental factors.	Emerging evidence; further investigation needed into specific hormonal pathways affected.	(Close, 2012; Reiter et al., 2009; Woldanska‐Okonska, 2014; Z. Yang et al., 2018)
Immune System	Upregulation of inflammatory markers (e.g., sICAM‐1, sVCAM‐1) associated with solar and geomagnetic activity.	Increased risk for inflammatory diseases and cardiovascular complications.	Age, pre‐existing inflammatory conditions, geographic location.	Moderate evidence; associations observed but require more comprehensive studies.	(Benassi et al., 2016; Lopez‐Martin et al., 2023; Uga et al., 2024)
Cognitive Function	Fluctuations in cognitive performance and increased reports of anxiety during geomagnetic storms.	Impacts on daily functioning and quality of life; potential exacerbation of existing cognitive impairments.	Pre‐existing cognitive conditions, environmental stressors, age.	Emerging evidence; relationships suggested but not well established.	(Krylov, 2017; Liddie et al., 2024)

*Note.* This table summarizes the documented effects of aurora‐related GMDs on different bodily systems, including on cardiovascular, nervous, endocrine, immune, and cognitive functions. Each row outlines the specific effects observed, their implications for health, influencing risk factors and confounders, the level of evidence from current research, identified research gaps, and references to original studies. The information highlights the potential health risks associated with geomagnetic activity (GMA) and emphasizes the need for further investigation into the underlying mechanisms and long‐term impacts on human health.

**Table 2:** Neurological and Psychiatric Health Impacts of Aurora‐Related Geomagnetic Disturbances

Neurological/Psychiatric disease	Effect of aurora‐related geomagnetic disturbances	Implications	Influencing risk factors/Effect modifiers and confounders	Level of evidence with current research	References
Depression and Mental Disorders	Time‐series and ecological analyses report increased psychiatric hospital admissions and suicide rates during periods of elevated GMA, with some data sets demonstrating seasonal variation and sex‐specific effects.	Heightened risk of psychiatric crises during geomagnetic storms.	Prior mental health conditions, stress levels, geographic location (higher latitudes).	Moderate evidence; studies show significant correlations but require more causal analysis.	(Berk et al., 2006; Kay, 1994; Nishimura et al., 2020; Sorsdahl et al., 2010)
Epilepsy	Increased frequency of seizures reported during periods of high geomagnetic activity.	Potential for exacerbated seizure control issues in patients with epilepsy.	Type of epilepsy, medication adherence, environmental factors.	Moderate evidence; some studies indicate a link but are limited in scope.	(Fournier, 2019; Guo et al., 2020; Persinger et al., 2005; Stoupel, 2002)
Anxiety Disorders	Fluctuations in anxiety levels correlated with geomagnetic activity changes.	Impacts on daily functioning and quality of life for affected individuals.	Pre‐existing anxiety disorders, environmental stressors, geographical factors.	Emerging evidence; the relationship is suggested but not well established.	(Krylov, 2017; Liddie et al., 2024)

## 3 Comprehensive Health Impacts of Geomagnetic Activity

GMA, particularly during periods of solar storms, has been linked to a wide range of adverse health impacts across various bodily systems.

### 3.1 Cardiovascular System Impact

There is a cumulative body of evidence linking GMDs with adverse cardiovascular outcomes (Borchert et al., 2022; Gmitrov & Gmitrova, 2004; Kiznys et al., 2020; Papailiou et al., 2010; Vanagaitė et al., 2022; Vencloviene, 2013). A study by Stoupel et al. found that periods of increased GMA correlated with higher rates of myocardial infarction, particularly in high‐risk individuals–such as those with existing heart disease (Stoupel, 2002). These effects are thought to be mediated, in large part, by alterations in HRV, which reflects ANS function. Decreased HRV during GMDs can lead to arrhythmias, elevated BP, and an increased risk of cardiovascular events, particularly in vulnerable populations such as the elderly and individuals with hypertension or coronary artery disease (Baevsky et al., 1997; Chernouss et al., 2001). A poor cardiac outcome has been associated with oxidative stress and inflammatory reactions via the NFKβ/TLR4 pathway, particularly following exposure to intense GMA, as compared to weak activity. In contrast, (Chang et al., 2024) point out that weak GMA might improve myocardial outcome by decreasing cytokines and augmenting the activity of superoxide dismutas, as evaluated in a myocardial ischemia/reperfusion injury (MI/RI) rat model subjected to different levels of GMA exposure.

### 3.2 Impact on Endocrine System

The endocrine system has been reported to be particularly vulnerable to heightened GMA, particularly in the regulation of stress and sleep‐related hormones. Melatonin, which regulates the circadian rhythm and has neuroprotective properties, is reported decreased during periods of high GMA (Rapoport et al., 2001). Such a reduction in melatonin has been linked to sleep disturbances, increased oxidative stress, and a higher risk of mood disorders, in particular depression and anxiety (Reiter et al., 2009). Cortisol, another critical hormone potentially affected by GMDs, is associated with the body's stress response. Elevated cortisol levels during solar storms have been observed, which may contribute to increased stress, anxiety, and a compromised immune function (Oraevskiĭ et al., n.d.). Geomagnetic storms can potentially impact the hypothalamic‐pituitary axis, thereby altering the secretion of several vital hormones (Breus et al., 2015). Woldańska‐Okońska and Czernicki (Woldańska‐Okońska & Czernicki, 2014) studied the effect of low frequency pulsating magnetic fields on hormonal secretion and discovered variations in prolactin, testosterone and estradiol in their patients; however, the difference in intensity of therapeutic magnetic fields and geomagnetic storms must be taken into consideration.

### 3.3 Immune and Inflammatory Responses

GMDs have also been linked to inflammatory responses, increasing levels of pro‐inflammatory cytokines and markers of oxidative stress and calcium (Leu, 2025). Such a pro‐inflammatory state can exacerbate chronic conditions, like rheumatoid arthritis, asthma, and other inflammatory diseases (Chen et al., 2012). Furthermore, studies suggest that GMDs can potentially compromise the immune system, reducing the body's ability to fight infections and potentially increasing its susceptibility to autoimmune diseases (Benassi et al., 2016).

### 3.4 Gastrointestinal System Impacts

The ANS's regulation of the gastrointestinal system may also be disrupted by GMDs. Altered gut motility, an imbalance in gut microbiota, and changes in digestive processes have been reported during periods of increased GMA, with symptoms including nausea, altered bowel habits, and irritable bowel syndrome exacerbations (Luo et al., 2021). These effects could potentially be more pronounced in individuals with pre‐existing gastrointestinal conditions or autonomic dysfunction, and can be particularly disruptive to the elderly.

The most important evidence for the above is summarized in Table 1.

### 3.5 Impact on Neurological Health

**Figure 1:**
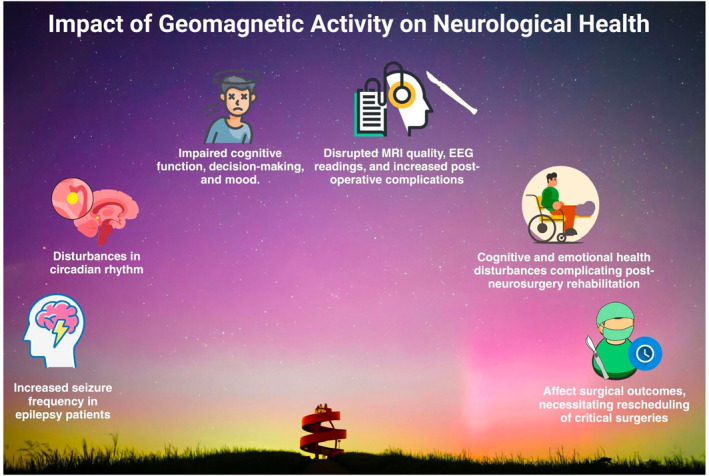
Potential Impacts of Geomagnetic Activity on Neurological Health. *Image sources*: Segments of the figure were created using visuals from Pexels, Flaticons, Freepik, Veryicon, and Publicdomainvectors, all licensed under Creative Commons.

While GMDs affect multiple physiological systems, their impact on neurological health is perhaps the most concerning. The brain's sensitivity to external electromagnetic changes, particularly those caused by GMDs, has been documented to disrupt electrical activity, neuronal communication, and brain rhythms (Allahverdiyeva & Allahverdiyev, 2022; Allakhverdiev et al., 2020; Novik & Smirnov, 2013). These disruptions can have wide‐ranging consequences for cognitive function, mood stability, and neurodegenerative disease progression (Figure 1), again with the elderly being most vulnerable.

### 3.6 Melatonin Dysregulation and Sleep Disorders

Melatonin levels decline during periods of heightened GMA (Persinger, 1995). This effect might be attributed to an impact on nitric oxide processes, thereby altering melatonin production in the pineal gland (Iashmanov & Koshelevskiǐ, 2008). Melatonin is not only essential for sleep regulation but also acts as a neuroprotective agent, as part of the homeostatic system to defend the brain from oxidative stress and inflammatory damage (Jammoul & Lawand, 2021). Reduced melatonin levels due to GMDs can potentially exacerbate sleep disorders, such as insomnia, and contribute to psychiatric conditions, like depression and anxiety, which are closely linked to circadian rhythm disruptions (Bhattacharyya et al., 2008; Martel et al., 2023). In rats subjected to chronic unpredictable mild stress to induce depression, exposure to high intensity GMA resulted in reductions in melatonin levels, biochemical markers of melatonin synthesis and melatonin 1 receptor expression, and intensified depression (Sumsuzzman et al., 2021). In contrast, mild/moderate GMA exposure mediated the opposite actions to thereby reduce biomarkers of depression (Sumsuzzman et al., 2021). Moreover, consequent to the reported neuroprotective role of melatonin, individuals suffering from neurodegenerative disorders, epitomized by Alzheimer's disease, could potentially experience accelerated cognitive decline due to potential loss of melatonin in slowing disease progression, and on associated sleep dysregulation and related impairments (Sumsuzzman et al., 2021) in areas associated with high GMA exposure.

### 3.7 Autonomic Nervous System (ANS) and Neurological Stress

GMDs have been reported to influence the ANS, which regulates involuntary functions, such as heart rate, digestion, and stress responses. As noted, variations in HRV have been observed during periods of intense GMA, with reduced HRV being associated with increased stress and anxiety levels (Zilli Vieira et al., 2022). This link between ANS function and psychiatric health suggests that GMDs may contribute to the exacerbation of anxiety disorders, particularly in those predisposed to mental health conditions (Krylov, 2017), as well as in those with less homeostatic compensatory mechanisms–such as in the elderly. Moreover, the impact of GMDs on HRV could also extend to cognitive function, as chronic stress is a well‐known contributor to cognitive decline and memory impairment.

### 3.8 Oxidative Stress and Neuroinflammation

GMA has been shown to increase oxidative stress (Kuleshova & Pulinets, 2000; Tian et al., 2024) systemically, but particularly in the brain, where it can lead to neuroinflammation. Oxidative stress is a significant contributor to the development of neurodegenerative diseases, including AD, Parkinson's disease, multiple sclerosis, and amyotrophic lateral sclerosis. In this regard, during geomagnetic storms, elevated levels of reactive oxygen species and inflammatory cytokines have been observed, both of which are known to induce neuronal cell damage and exacerbate both systemic and neuroinflammatory conditions. This oxidative burden not only accelerates the aging process in the brain but can also contribute to the development of mood disorders, such as depression, which has likewise been linked to chronic brain inflammation (Zhang et al., 2017). Additionally, Zhang et al. noted in their research on human neuroblastoma cells that shielding of geomagnetic fields can reduce the production of hydrogen peroxide and decreases superoxide dismutase (Zhang et al., 2017).

### 3.9 Seizures and Neuronal Excitability

Evolving research has shown that changes in GMA can influence neuronal excitability, leading to an increased risk of seizures and other neurological events. During periods of intense solar storms, an uptick in seizure frequency has been observed in patients with epilepsy, potentially due to the altered electrical balance in the brain caused by GMDs (Stoupel, 2002). In parallel, experimental work by Michael Persinger demonstrates that weak, patterned magnetic fields can modulate temporal–limbic activity, inducing epileptiform EEG changes and experiential phenomena resembling temporal lobe lability. Animal studies further suggest that specific low‐intensity field configurations may alter seizure thresholds, supporting the concept that environmental electromagnetic influences operate along a continuum of temporal lobe excitability. Together, these findings provide a mechanistic framework linking external electromagnetic variability with seizure susceptibility. Additionally, individuals prone to migraines report increased incidences of headaches during periods of high GMA, further suggesting that GMDs may influence brain electrical activity and neuronal excitability (Milojević, 2016).

The neuropsychiatric implications of aurora‐associated GMDs are summarized in Table 2. Importantly, this framework extends beyond periods of overt auroral visibility to encompass the broader spectrum of heightened GMA. Aurora‐associated GMDs represent the upper end of this spectrum—typically reflecting intensified magnetospheric–ionospheric interactions and increased electromagnetic variability—but similar, albeit subtler, perturbations in geomagnetic fields occur during non‐auroral intervals. Emerging evidence suggests that even these sub‐auroral fluctuations may influence neuronal excitability, circadian regulation, and autonomic balance, thereby contributing to variability in seizure susceptibility, mood disorders, and other neuropsychiatric outcomes. Accordingly, the associations outlined in Table 2 should be interpreted within a continuum model, wherein both pronounced (aurora‐associated) and moderate elevations in GMA may exert biologically relevant effects, differing primarily in magnitude rather than in fundamental mechanism.

## 4 Vulnerable Populations, the Elderly and Neuropsychiatric Outcomes

Vulnerable populations, including the elderly, individuals with pre‐existing psychiatric or neurodegenerative conditions, and those with autonomic dysfunction, may be particularly susceptible to the health impacts of GMDs. In older adults, the combination of reduced melatonin production, increased oxidative stress, and impaired autonomic function can accelerate cognitive decline and exacerbate mood disorders such as depression and anxiety (Liddie et al., 2024). GMAs, largely associated with solar events and, in particular with coronal mass ejections, and readily noticeable as visual auroras, can have subtle but, nevertheless, significant health impacts, particularly on the elderly. Unlike the general population, older adults are more vulnerable consequent to their greater predisposition to cardiovascular issues, particularly arrhythmias and heart attacks, which have been correlated with GMDs. Research, especially from regions like Russia and Eastern Europe, has shown spikes in hospital admissions and mortality among the elderly during geomagnetic storms, suggesting a real, albeit still debated, link between space weather and human health. Given these patterns, heightened awareness and monitoring of GMA could assist preventive care strategies for aging populations, potentially mitigating adverse effects during periods of unusually high solar activity. Furthermore, individuals with epilepsy or migraines may experience more frequent and severe episodes during periods of heightened GMD, necessitating extra precautions and/or treatments (D’Agnano et al., 2024).

Additionally, individuals with pre‐existing psychiatric conditions, particularly those suffering from anxiety disorders or bipolar disorder, may find their symptoms exacerbated during periods of geomagnetic unrest. Given the growing body of evidence linking GMDs to increased stress and anxiety levels, healthcare providers should consider GMA as a potential contributing factor when treating psychiatric patients, especially during periods of solar storms (Erendjenov, 2024; He et al., 2025; Liddie et al., 2024; Maryankin, 2024; Noskov et al., 2024; Oskolkova et al., 2023; Preka‐Papadema & Tzanis, 2024; Ragozin et al., 2025).

## 5 Impacts of Aurora‐Related Geomagnetic Activity on Neurosurgery

GMA during aurora events, especially in 2024, has been observed well beyond the polar regions, affecting areas across the world beyond the traditional ones (Liddie et al., 2024). This widespread occurrence has led to greater concern about its effects on human health, particularly in sensitive medical fields like neurosurgery. Recent studies have linked GMA to diverse physiological, psychological, and cognitive disturbances (Tables 1 and 2), all of which may pose risks to neurosurgical patients. Although most research has largely centered on cardiovascular and psychological effects, neurosurgery patients represent a unique population worthy of further examination.

## 6 System Level Disruptions

### 6.1 Physiological Effects on Patients

*Increased Seizure Activity*: Heightened GMA has been associated with increased seizure frequency in epilepsy subjects as evident in certain human (Persinger & Psych, 1995) and mice model studies) (Persinger, 1995; Persinger & Psych, 1995). In this regard, research indicates that periods of high GMA correlate with a notable rise in seizure activity among those with pre‐existing neurological vulnerabilities, necessitating more intensive monitoring and surgical planning during aurora‐related events (Liddie et al., 2024).

*Circadian Rhythm Disruption*: Circadian rhythm disturbances due to GMA can disrupt melatonin production, leading to poor sleep quality (Close, 2012; Martel et al., 2023; Xue et al., 2021; Yamshanov & Koshelevskii, 2013). Studies have demonstrated that altered sleep cycles can significantly impact post‐operative recovery in neurosurgical patients, prolonging hospital stays and increasing the risk of complications (Albayrak et al., 2025; Kovoor et al., 2022; Rosenberg‐Adamsen et al., 1996; Xue et al., 2021; B. Yang et al., 2025). Such disruptions can be particularly detrimental to patients recovering from brain surgeries, where sleep is considered crucial to the healing and recovery processes.

*Anxiety and Stress*: Geomagnetic storms are, as noted, also associated with elevated anxiety levels, which may exacerbate stress in patients undergoing neurosurgical procedures. Periods of high GMA have been shown to intensify anxiety symptoms across both pre‐ and post‐operative neurosurgical patients, with the potential to impact recovery outcomes and overall prognosis (Krylov, 2017).

### 6.2 Cognitive and Behavioral Changes

*Cognitive Variability*: Geomagnetic disturbances have been reported to temporarily affect cognitive function, impacting patient readiness for surgery and possibly complicating consent processes. Specifically, fluctuations in GMA can impair cognitive performance, potentially interfering with decision‐making in neurosurgical contexts, where mental clarity is essential for both patients and medical professionals (Liddie et al., 2024).

*Psychiatric Effects*: GMA has been associated with mood changes, particularly with regards to increased rates of depression and irritability. Studies have linked GMDs with psychiatric hospital admissions, particularly among vulnerable populations, which may complicate post‐operative psychiatric outcomes for neurosurgical patients (Krylov, 2017). Table 3 explores the key studies evaluating the influence of geomagnetic waves on human physiological and behavioral parameters.

**Table 3:** Key Studies Investigating the Influence of Geomagnetic and Helio‐Magnetic Activity on Human Physiological and Behavioral Parameters

Author	Type of study	Patient population	Disease/Condition studied	Results	Implications	References
Mattoni et al.	Longitudinal observational study	20 healthy participants from UNC‐Chapel Hill	Geomagnetic activity's effect on heart rate variability (HRV)	Significant correlations were found between HRV components and geomagnetic/solar activity initially, but only minor effects remained after statistical corrections.	Prior findings linking geomagnetic activity to HRV may be overstated; findings suggest only a minor effect, highlighting the need for more rigorous statistical analysis and larger studies.	Mattoni et al. (2020)
Feigin et al.	Time‐stratified case‐crossover study	11,453 stroke patients across New Zealand, Australia, UK, France, and Sweden (1981–2004)	Stroke	Geomagnetic storms increased the risk of stroke by 19% overall, with up to a 52% increase in patients under 65 during severe storms.	Geomagnetic activity may be an environmental risk factor for stroke. Monitoring geomagnetic storms could help with stroke prevention strategies, especially in high‐risk groups.	Feigin et al. (2014)
Weydahl et al.	Observational study	25 healthy subjects in Alta, Norway (70°N latitude)	Influence of geomagnetic activity on melatonin secretion	Significant changes in geomagnetic activity (>80 nT/3h) were associated with a decrease in salivary melatonin concentration. Circadian and circannual rhythms were found for both melatonin and geomagnetic activity.	Geomagnetic disturbances may influence melatonin secretion, particularly in high‐latitude regions where geomagnetic activity is pronounced. Further research could explore implications for sleep and circadian health.	Weydahl et al. (2000)
Thoss et al.	Observational study with field manipulation	55 healthy human volunteers	Influence of geomagnetic field on visual sensitivity	The geomagnetic field increased light sensitivity by 6%–7%, with reduced perceived background luminance by 10%–15% when the magnetic field was aligned with the viewing direction.	Geomagnetic fields may subtly affect human visual perception, likely through a radical‐pair mechanism. This could have implications for understanding vision under natural magnetic conditions.	Thoss and Bartsch (2007)
Dimitrova et al.	Observational study	86 volunteers at middle latitudes	Influence of geomagnetic storms on arterial blood pressure (BP) and heart rate	Systolic and diastolic BP increased significantly from the day before to the second day after a geomagnetic storm, regardless of sex or medication.	Geomagnetic storms may influence blood pressure, with potential implications for cardiovascular health monitoring during periods of high geomagnetic activity.	Dimitrova et al. (2004)
Berk et al.	Observational study analyzing national data	National suicide data for 51,845 males and 16,327 females in Australia (1968–2002)	Impact of geomagnetic storms on suicide rates	Female suicide rates increased significantly during geomagnetic storms in autumn (*P* = 0.01), while no significant effect was found in males (*P* = 0.16).	Geomagnetic storms may influence behavior and mood, especially in females, highlighting a potential public health concern regarding environmental EMF exposure.	Berk et al. (2006)
Otsuka et al.	Observational study	Eight healthy subjects in Alta, Norway (subarctic region, 70°N)	Impact of geomagnetic disturbances on heart rate variability (HRV)	During high geomagnetic disturbance days, heart rate increased by 5.9% (*P* = 0.020) and HRV decreased by 25.2% (*P* = 0.002), with lower spectral power mainly at frequencies <0.04 Hz.	Geomagnetic disturbances may reduce HRV, affecting autonomic regulation, which could influence cardiovascular health in subarctic regions.	Otsuka et al. (2000)
Watanabe et al.	Longitudinal observational study	Single clinically healthy cardiologist, monitored over 11 years	Influence of helio‐ and geomagnetic activity on cardiovascular parameters	Heart rate showed a direct, solar cycle stage‐dependent association with Wolf numbers (WN), while HRV and blood pressure variability had inverse relationships with WN. Coherent HR changes with WN occurred at ∼7.33‐month intervals.	Findings suggest a link between solar and geomagnetic activity and human cardiovascular function, highlighting potential long‐term impacts on circulation and heart health.	Watanabe et al. (2000)

### 6.3 Surgical Procedures and Techniques

*Imaging and Navigation Systems*: Advanced neurosurgical procedures heavily rely on imaging techniques such as magnetic resonance imaging, which can be affected by GMDs (Sarimov et al., 2005). Research indicates that geomagnetic storms lead to fluctuations in magnetic field strength (Kasper et al., 2015). This may theoretically introduce magnetic field variability relevant to imaging systems and, if so, would complicate surgical planning ‐ although direct clinical evidence remains limited. The precision of imaging tools is critical for neurosurgical interventions, where even slight inaccuracies can lead to significant risks.

*Electroencephalography (EEG)*: EEG readings, essential for monitoring brain activity during neurosurgery, can be disrupted by GMA (Marasanov et al., 2021). Environmental electromagnetic fields during geomagnetic storms introduce noise into EEG signals, making it difficult to interpret results and potentially leading to misdiagnosis or improper surgical interventions (Kanunikov & Kiselev, 2014).

### 6.4 Post‐Operative Care and Monitoring

*Complication Monitoring*: Post‐operative monitoring is clearly essential for neurosurgical patients, and should be especially considered during GMDs. In this regard, studies report that GMA increases the risks of complications, such as heightened pain perception and fluctuations in BP, which are important concerns during post‐operative management (Cornélissen et al., 2002). This demands heightened vigilance from healthcare providers during intensified aurora‐related disturbances.

*Rehabilitation Challenges*: Cognitive and emotional health can, as noted, be significantly affected by GMA, thereby complicating rehabilitation efforts after neurosurgery. Tailored rehabilitation strategies that account for potential cognitive disruptions caused by heightened GMA during recovery periods are essential.

### 6.5 Implications for Surgical Scheduling

*Timing of Procedures*: Historical data suggest a correlation between GMA and patient outcome emphasizing the need to consider GMA when scheduling elective surgeries (He et al., 2025). Neurosurgeons may need to avoid scheduling critical surgeries during periods of particularly high GMA to minimize potential risks and optimize recovery.

Aurora‐related GMA presents a unique series of challenges to neurosurgery. The physiological, cognitive, and emotional effects of GMA can significantly affect surgical planning, execution, and post‐operative care. Neurosurgeons need to be aware of these influences and incorporate GMA monitoring into patient care protocols. As the frequency of aurora‐related GMA increases globally, understanding its full impact on neurosurgery becomes more essential. Further research is hence needed to develop evidence‐based guidelines to manage the noted associated risks effectively.

## 7 Recommendations for Future Research and Public Health Implications

**Figure 2:**
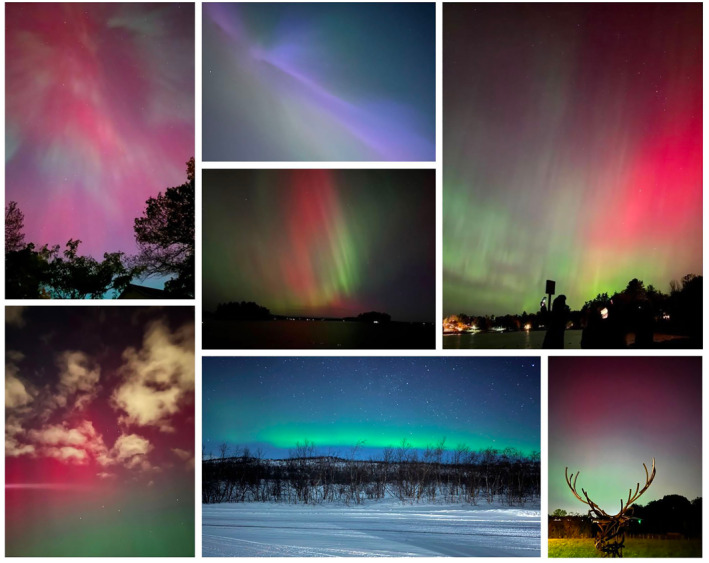
A collage of recent Aurora Borealis displays from around the world. These images showcase the vibrant, dancing lights of the northern skies captured in Canada, the United Kingdom, and Norway. Each location offers a unique perspective on the auroras' colors and patterns, shaped by the local geography and atmospheric conditions.

Given the increasing prevalence of auroras and the corresponding rise in GMDs worldwide (Figure 2), it is essential for future research to focus on identifying the full scope of health impacts, particularly on the nervous system. Future studies should aim to investigate the long‐term effects of repeated exposure to GMDs, especially in relation to cognitive function, mood and neurodegenerative diseases. Furthermore, research should prioritize understanding the impacts on vulnerable populations, including the elderly and individuals with pre‐existing conditions, to develop monitoring procedures as well as targeted preventive strategies and therapeutic interventions. The relationship between GMA and neurosurgical outcomes is an emerging area of research interest, with much still unknown. Future studies should best focus on large‐scale, multi‐center trials to gain a greater understanding of how GMA impacts neurosurgery patients, indeed all major surgical procedures, and to refine surgical practices accordingly. Could longitudinal cohort studies on surgical outcomes during GMDs and/or randomized trials of targeted interventions prove beneficial? Additionally, research should aim to uncover the biological and molecular mechanisms behind GMA's influence on neurological health, particularly in relation to post‐operative recovery, cognitive function, and seizure activity.

Additionally, given the cyclical nature of solar and GMA, which follows an approximately 11‐year solar cycle, it is important to consider periods of heightened GMD within these predictable temporal patterns when evaluating potential public health implications (Zilli Vieira et al., 2019). Efforts should be made to raise awareness of the health risks associated with GMDs, particularly for vulnerable groups such as the elderly, and healthcare professionals should be educated on how to monitor and mitigate these risks during periods of heightened GMA.

## 8 Limitations of Current Research

Although there is growing evidence supporting the connection between GMDs and health impacts, significant gaps remain in our understanding of these phenomena. Much of the current research relies on observational, animal and experimental laboratory studies which are inherently limited by their inability to establish causality. Furthermore, studies examining the health impacts of GMDs often suffer from small sample sizes, variabilities in study designs and a lack of controlled environments (e.g., geographic locations, ambient light, seasonal changes), which makes it difficult to isolate the effects of GMA from other confounding factors. Moreover, the variability in individual susceptibility to GMDs remains poorly understood, and future research should aim to uncover the genetic or physiological factors that contribute to this variability.

Finally, the underlying biological molecular mechanisms that underpin the reported health impacts of GMDs remain largely underexplored. For example, how do geomagnetic fluctuations specifically disrupt neuronal excitability or melatonin regulation? How could potential pathways (e.g., oxidative stress, circadian rhythm disruption) be explored in animal models in a translationally relevant manner? Which biological markers could potentially best be followed in humans?

## 9 Conclusion

GMDs have reported wide‐reaching health impacts, influencing multiple physiological functions, including the cardiovascular, endocrine, immune, and gastrointestinal systems. However, the neurological and neuropsychiatric effects of GMDs remain an under‐explored area of research, despite growing evidence that GMA may contribute to sleep disturbances, cognitive decline, neurodegenerative disease progression, and mood disorders. As auroras become more frequent in regions outside the polar areas, exposure to GMDs is increasing, necessitating greater awareness and further research into their health impacts and particularly into the molecular mechanisms that underpin them.

## Declaration of Generative AI in Scientific Writing

The authors declare that AI and AI‐assisted technologies were NOT used in the writing/preparation of this manuscript.

## Conflict of Interest

The authors declare no conflicts of interest relevant to this study.

## Data Availability

This is a “review article” which provides an evaluation of the available published scientific literature, as detailed in Section 2 (Material and Methods)–initial paragraph. Consequently, and in line with most review articles, this does not contain any “primary data” (as routinely found in “original research articles”). Notably, the published studies evaluated in this review are noted in the text and Tables (as they appear) and are fully referenced at the end of this article.
